# Creating Virtual Community For Older Adults: During the COVID-19 Pandemic And Beyond

**DOI:** 10.1093/geroni/igab046.364

**Published:** 2021-12-17

**Authors:** Lawrence Kosick, Jennifer Hunt, Erica Solway

## Abstract

Combining data on health and well-being from the University of Michigan National Poll on Healthy Aging (NPHA) with case studies and data from GetSetUp, a virtual online learning community, and the Michigan Department of Health and Human Services (MDHHS), this symposium will highlight how virtual community can be created and supported during the COVID-19 pandemic and beyond. Polling data on loneliness and physical environments demonstrate the need for opportunities for connection before and during the pandemic. Other polling data from the NPHA shows telehealth visits increased significantly as did the use of video chat technology. These findings suggest that comfort with technology may help support aging in place. GetSetUp helps to make this possible with customized learning to help older adults overcome hurdles to tech adoption and use. GetSetUp classes focus on supporting social connection and providing information on resources and services. Beyond the pandemic, these services will remain critical for many older adults, including those facing mobility limitations, those with limited community, and those looking to diversify their networks. The Senior Deputy Director of Aging and Adult Services Agency will highlight how Michigan combines data and technology to support Michigan’s aging network. The GetSetUp and MDHHS virtual community allowed for a statewide connection to health and aging services, including programs such as vaccine information sessions. The data and case studies described will highlight the need for connection during the COVID-19 pandemic and how a startup and State worked together to address this need.

